# Coupling of carbon and silicon geochemical cycles in rivers and lakes

**DOI:** 10.1038/srep35832

**Published:** 2016-10-24

**Authors:** Baoli Wang, Cong-Qiang Liu, Stephen C. Maberly, Fushun Wang, Jens Hartmann

**Affiliations:** 1 Institute of Surface-Earth System Science, Tianjin University, Tianjin 300072, China; 2 State Key Laboratory of Environmental Geochemistry, Institute of Geochemistry, Chinese Academy of Sciences, Guiyang 550002, China; 3 Centre for Ecology & Hydrology, Lancaster Environment Centre, Library Avenue, LA1 4AP Bailrigg, United Kingdom; 4 Institute of Applied Radiation, School of Environmental and Chemical Engineering, Shanghai University, Shanghai 200433, China; 5 Institute for Geology, Center for Earth System Research and Sustainability (CEN), Universität Hamburg, Hamburg 20146, Germany

## Abstract

Carbon (C) and silicon (Si) biogeochemical cycles are important factors in the regulation of atmospheric CO_2_ concentrations and hence climate change. Theoretically, these elements are linked by chemical weathering and organism stoichiometry, but this coupling has not been investigated in freshwaters. Here we compiled data from global rivers and lakes in the United States of America and the United Kingdom, in order to characterize the stoichiometry between the biogeochemical cycles of C and Si. In rivers this coupling is confirmed by a significant relationship between HCO_3_^−^/Na^+^ and DSi/Na^+^, and DSi:HCO_3_^−^ ratio can reflect the mineral source of chemical weathering. In lakes, however, these characteristic ratios of chemical weathering are altered by algal activity. The lacustrine Si:C atomic ratio is negative feedback regulation by phytoplankton, which may result in this ratio in algal assemblages similar to that in water column. And this regulation suggests lacustrine photosynthetic C fixation in this equilibrium state is quantitative and depends on the DSi concentration. These findings provide new insights into the role of freshwaters in global C and Si biogeochemical cycles.

Chemical weathering of silicate minerals consumes atmospheric CO_2_ and stoichiometrically produces dissolved silica (DSi, referred to silicic acid, H_4_SiO_4_) and HCO_3_^−^ 1,2. These solutes are transported to the coastal zone and lakes by rivers where the carbon can be synthesized into organic matter by phytoplankton and the silica can be used to produce diatom frustules. Ultimately, some of these organisms sink to the bottom sediments where carbon (C) and silicon (Si) can be sequestered. Thus, silicate weathering has a net-sink effect on atmospheric CO_2_^1^,^3^. However, this is not the case for carbonate mineral weathering by carbonic acid, because CO_2_ is re-released to the atmosphere when carbonate mineral is re-precipitated on geological time scales (>10 to 100 ka). Besides, strong acids (i.e. sulfuric and nitric acid) from anthropogenic sources have enhanced carbonate weathering and CO_2_ evasion^4^,^5^,^6^. Therefore, C and Si biogeochemical cycles are important in regulating atmospheric CO_2_ concentrations and hence climate change.

Since the industrial revolution, anthropogenic perturbation such as nutrient enrichment and construction of dams have altered chemical weathering and C and Si fluxes through the river systems^7^,^8^, and the related mechanisms are suggested to further research^9^. C sequestration coupled with the Si cycle in agricultural ecosystems has recently been summarized^10^, however, coupling of C and Si geochemical cycles have not yet been highlighted in freshwaters. This may be because (1) riverine inorganic C and dissolved Si (DSi) concentrations vary greatly on a global scale^11^ and there is not a conspicuous relationship between chemical species such as calcium and bicarbonate^12^, (2) C and Si are traditionally considered to be less important than nitrogen (N) and phosphorus (P) in limiting the growth of photosynthetic organism.

Nutrient biogeochemical cycles in aquatic ecosystem linked through the metabolic activity of living organisms^13^,^14^. For example, diatoms require DSi to build their frustules^15^ and C for their organic molecules. To test the extent of coupling between C and Si in inland waters, we compiled data from global rivers, and lakes from the United States of America and the English Lake District. The specific aims of this study were to (1) characterize the relationship between dissolved inorganic C and Si in rivers and (2) determine how phytoplankton couples the C and Si biogeochemical cycles in lakes.

## Results

### Variation of carbon and silicon in rivers

**Figure 1:**
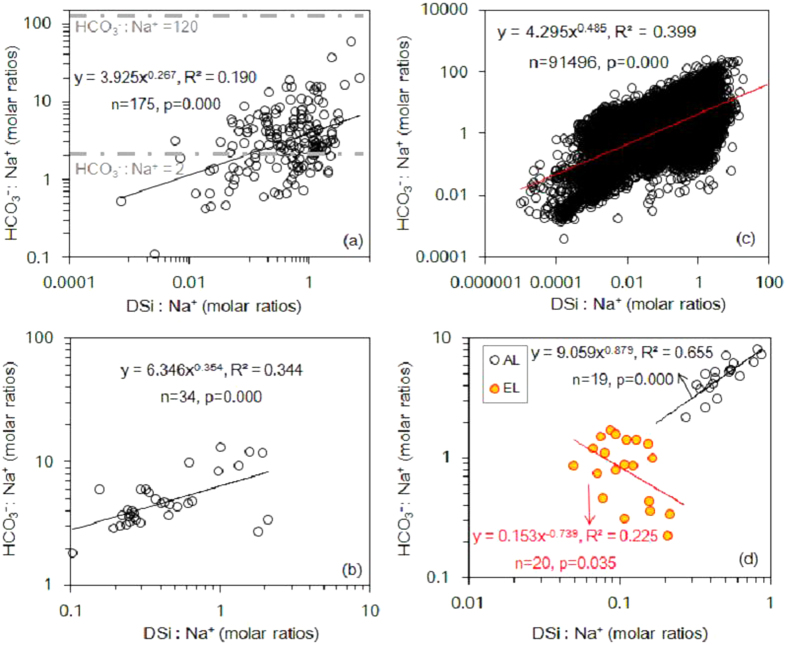
Relationships between Na-normalized DSi and HCO_3_^−^. (**a**) From the 175 rivers; (**b**) From the Changjiang River; (**c**) From the GLORICH database (Supplementary Methods); (**d**) From the lakes in United States of America (AL) and in the English Lake District (EL). The data from the American Lakes are the mean of each dataset (Supplementary Table 2), whereas the data from the English Lake District are the mean for each lake every five years between 1991 and 2010 (Supplementary Methods).

**Figure 2:**
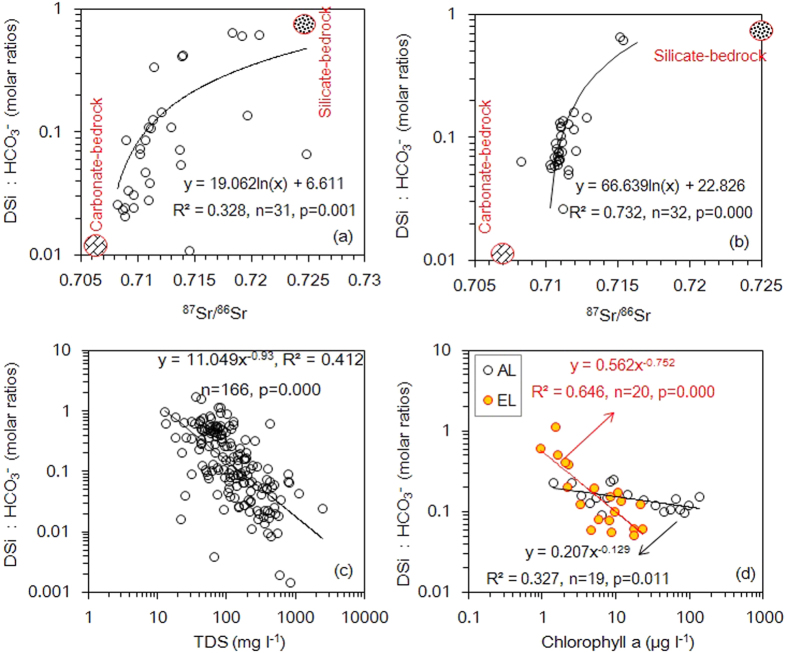
Relationship between DSi:HCO_3_^−^ and ^87^Sr/^86^Sr, TDS (total dissolved solid), and chlorophyll *a*, respectively. Data in (**a,c**) are from the 175 rivers; data in (**b**) are from the Changjiang River, data in (**d**) are from the two lake regions (referred to Fig. 1).

Major ion concentrations ranged over three orders of magnitude in the 175 global rivers (Supplementary Tables 4). The average concentrations of DSi and HCO_3_^−^ were 165 and 1296 μmol l^−1^, respectively. Sodium (Na)-normalized DSi and HCO_3_^−^ concentrations showed a significant positive correlation (Fig. 1a–c), however, this was not the case for their absolute concentrations in the 175 global rivers (Supplementary Table 7). As expected, Na-normalized DSi and HCO_3_^−^ ratios increased from watersheds dominated by silicate weathering to those dominated by carbonate weathering, while DSi:HCO_3_^−^ ratios showed the reverse tendency, suggesting that they can be used to discern the source of mineral weathering in global rivers (Figs 1a–c and 2a,b). DSi:HCO_3_^−^ ratios were positively correlated with ^87^Sr/^86^Sr (Fig. 2a,b) and negatively correlated with total dissolved solid (TDS) (Fig. 2c). DSi and HCO_3_^−^ in the Changjiang catchment showed a similar geochemical behaviour to those in the 175 global rivers (Figs 1a,b and 2a,b).

### Variation of carbon and silicon in lakes

**Figure 3:**
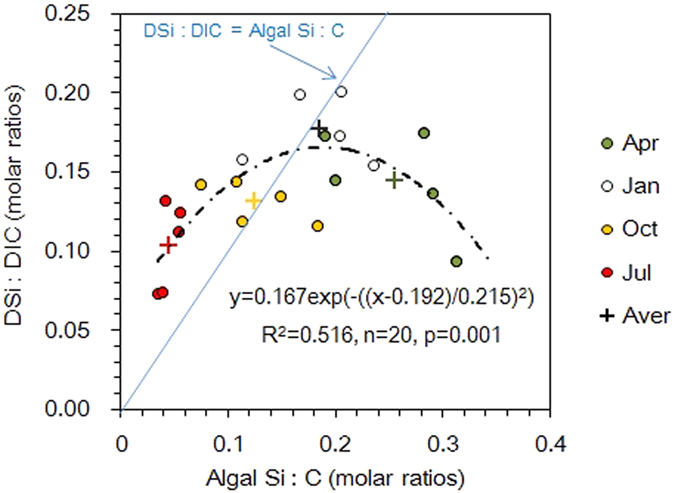
Relationship between the DSi:DIC and the algal Si:C ratios in the English Lake District. The data represent the means from January, April, July and October for 20 lakes every 5 years (1991, 1995, 2000, 2005, and 2010). The cross represents the seasonal means of the 20 lakes over 5 years.

Average DSi and HCO_3_^−^ concentrations in the American lakes were of a similar magnitude to those in the 175 global rivers, and an order of magnitude greater than the concentrations in the English Lake District (Supplementary Tables 4–6) because their catchments were small and mainly comprised slowly weathering igneous and silicate rocks. Average contribution of HCO_3_^−^ to dissolved inorganic carbon (DIC) in the English Lake District (78.8%) was less than that in the American lakes (91%) because of the lower HCO_3_^−^ concentrations. For both lake regions, both absolute and Na-normalized DSi and HCO_3_^−^ concentrations were correlated significantly, but the sign of the correlation (i.e. positive or negative) was different (Fig. 1d and Supplementary Tables 8 and 9). The DSi:HCO_3_^−^ ratio was negatively correlated to chlorophyll *a*, and this relationship was more significant in the English Lake District than in the American lakes (Fig. 2d), explaining the different sign of the correlation. In the English Lake District, the relationship between the DSi:DIC and the algal Si:C ratio varied throughout the year and took the form of a bell-shaped curve (Fig. 3).

## Discussion

Riverine DSi and HCO_3_^−^ mainly originate from chemical weathering. The variation in their concentration results from heterogeneity in factors such as land cover, runoff, and evaporation in different rivers at the global scale^11^, but Na-normalized concentrations can eliminate the influences of these factors as Na is mostly originating from weathering processes^16^,^17^. This is the reason that the Na-normalized DSi and HCO_3_^−^ ratios are linked and can reflect their origins. The similar tracing function of the DSi:HCO_3_^−^ ratio is supported by its significant relation to ^87^Sr/^86^Sr, which is known to identify the nature of watershed rock and local weathering processes^12^,^16^. ^87^Sr/^86^Sr is a better tracer of the end member of carbonate weathering than that of silicate weathering (Fig. 2a), as this ratio will depend on the age of the geological province^18^,^19^. The DSi:HCO_3_^−^ ratio is influenced by the contribution of silicate weathering, which in turn is strongly controlled by runoff^12^,^20^,^21^. Data from the 175 global rivers are consistent with this: the DSi:HCO_3_^−^ ratios were positively correlated with runoff (r = 0.373, n = 167, p < 0.001). The DSi:HCO_3_^−^ ratio was also negatively correlated with TDS (Fig. 2c) since the rate of silicate chemical weathering is a function of runoff and TDS^12^.

Lacustrine DSi:HCO_3_^−^ ratios do not reflect the characteristics of chemical weathering determined by the catchment because algal activity differentially takes up Si vs carbon and the ratio therefore decreases with an increase in phytoplankton biomass (i.e. chlorophyll *a*). Diatoms can use both CO_2_ and HCO_3_^−^ as an inorganic C source^22^. Thus, DSi:DIC ratio responds to algal activity more significantly than DSi:HCO_3_^−^ ratio, although HCO_3_^−^ is a dominant species in DIC. The average Si:C atomic ratio of freshwater diatoms, 0.79^23^, is usually larger than that of inflowing rivers. Therefore, with an increase in diatom biomass, residual solutes show decreasing Si:C atomic ratios^24^. This conclusion is also strengthened by the data from the English Lake District where the inflowing streams had average DSi and DIC concentrations of 43.8 and 370 μmol l^−1^ respectively (i.e. DSi:DIC = 0.12)^25^,^26^, whereas the average DSi and DIC concentrations in the lakes themselves were 22.3 and 243.9 μmol l^−1^ respectively (i.e. DSi:DIC = 0.09; Supplementary Tables 6).

**Figure 4:**
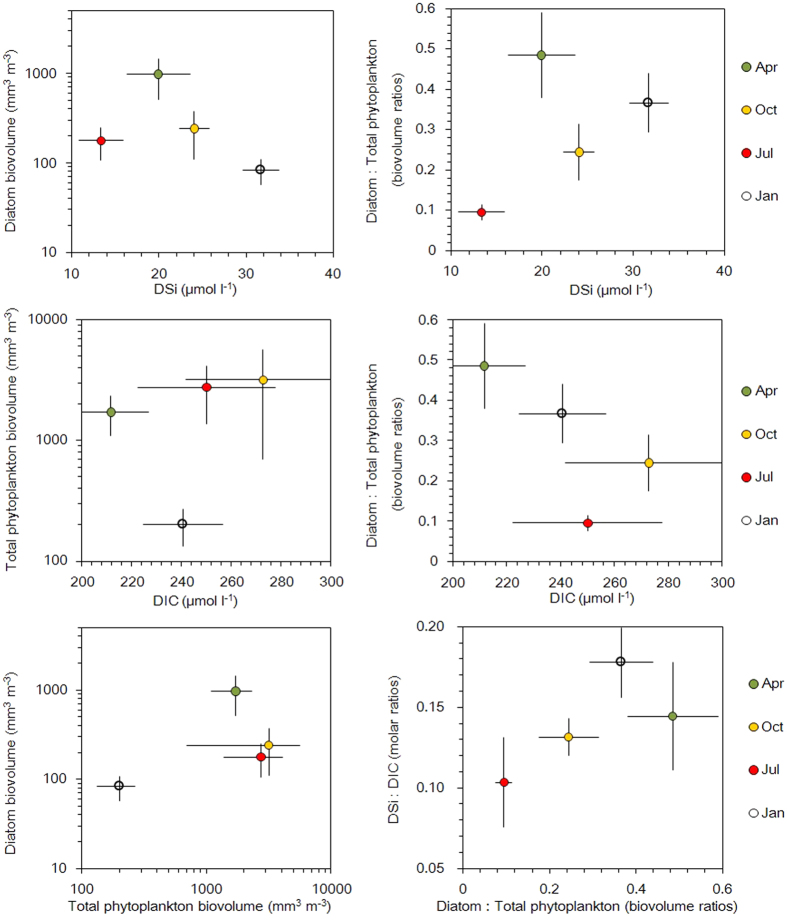
Relationships between selected variables of lakes in the English Lake District. The data represent the average and standard deviation of Jan, Apr, Jul and Oct for 20 lakes over 5 years. The standard deviation was calculated from the average of each year.

The long-term data from the English Lake District (1991–2010) provide further insights into the interactions between the DSi:DIC and the algal Si:C ratios (Figs 3 and 4). In winter, low temperature and light limits algal growth. In January, the algal assemblages have their lowest biovolume, of which diatoms represent 37%, and have an Si:C ratio similar to the water DSi:DIC ratio. In spring, diatoms bloom and dominate the phytoplankton (49% of total biovolume), resulting in an increase of the algal Si:C ratio and a decrease in the water DSi:DIC ratio. In summer, Si depletion, stratification and nutrient shifts favour non-diatom phytoplankton^27^. Although diatoms decrease, the total algal biovolume is higher than in spring and the algal Si:C and DSi:DIC ratios were at a minimum. In the autumn, continued inputs of Si from the catchment and a weakening of stratification, start to permit diatom growth again. This results in an increase in the algal Si:C ratio, bringing them into equilibrium with the water DSi:DIC ratio.

Overall, this bell-shaped relation through the year (Fig. 3) has an average DSi:DIC ratio of 0.14 that is approximately equal to the average algal Si:C ratio of 0.15, values very close to the well-known Redfield ratio, 106C:16N:16Si:1P^28^. This bell-shaped curve suggests that there is a negative feedback regulation on Si and C stoichiometry by lacustrine phytoplankton. When the algal Si:C value deviates from the bioavailable Si:C ratio, the relative contribution from diatoms changes to adjust for the deviation. This may bring the system into an equilibrium state, which means that the Si:C atomic ratio of the algal assemblage is similar to that in the water column (Fig. 3). It is likely that this stoichiometric homeostasis may apply to other nutrients in lacustrine system, such as N and P. Similar stoichiometric homeostasis of N and P ([NO_3_^−^]:[PO_4_^3−^]≈15:1) in the oceans is also thought to be regulated by algae^29^.

The coupling of Si and C in rivers reflects their origins and controls by chemical weathering, whereas in lakes this signature is modified by algal activity. Many human-induced environmental problems, such as climate change and eutrophication, are the result of disruptions of natural nutrient stoichiometry^14^. Our study supports this. Once lacustrine systems deviate from the equilibrium state, they are prone to algal blooms. Furthermore, our homeostatic theory derived from the English Lake District also suggests that in the equilibrium state, C fixation by algae in lakes is quantitative and depends on the DSi concentration, and this hypothesis maybe requires to be tested more widely with other data. These findings provide some new insights into global C and Si biogeochemical cycles.

## Methods

Data from global rivers were collected from the GEMS-GLORI world river discharge database^30^, from which 175 rivers with SiO_2_ (i.e. DSi) concentrations were selected (Supplementary Table 1). Data from small to medium-sized river catchments were collected from GLORICH database, and the samples having DSi, HCO_3_^−^ and Na concentrations were selected. Data from American lakes were derived from the 2007 National Lakes Assessment, and divided into 19 datasets according to chlorophyll *a* concentration (Supplementary Table 2). Surveys of the lakes in the English Lake District were conducted seasonally in 1991, 1995, 2000, 2005 and 2010, and chemical variables and phytoplankton species composition were investigated. Algae were identified and enumerated using a microscope^31^. The biovolume of each species was calculated geometrically^32^, and the C and Si contents per cell were calculated on the basis of the relationship between cell volume and cellular C and Si contents^33^,^34^, respectively. Concentrations of CO_2_, HCO_3_^−^ and CO_3_^2−^ were calculated based on alkalinity, pH and temperature, with equilibrium constants corrected for temperature and ionic strength^35^. Further details about the Methods are found in the Supplementary Methods.

## Additional Information

**How to cite this article**: Wang, B. *et al*. Coupling of carbon and silicon geochemical cycles in rivers and lakes. *Sci. Rep.* **6**, 35832; doi: 10.1038/srep35832 (2016).

## Supplementary Material

Supplementary Information
