# Supplementary material for: Coupling of carbon and silicon geochemical cycles in rivers and lakes
Source: Sci Rep. 2016 Oct 24;6:35832. doi: 10.1038/srep35832 (PMC5075770; doi:10.1038/srep35832)
Supplement: Supplementary Information [file srep35832-s1.doc]

**Supplementary Methods**

Baoli Wang^1, 2^, ^*^, Cong-Qiang Liu^2^, Stephen C. Maberly^3^, Fushun Wang^4, *^, Jens Hartmann^5^

^1^Institute of Surface-Earth System Science, Tianjin University, Tianjin 300072, China

^2^State Key Laboratory of Environmental Geochemistry, Institute of Geochemistry, Chinese Academy of Sciences, Guiyang 550002, China

^3^Centre for Ecology & Hydrology, Lancaster Environment Centre, Library Avenue, LA1 4AP Bailrigg, United Kingdom

^4^Institute of Applied Radiation, School of Environmental and Chemical Engineering, Shanghai University, Shanghai 200433, China

^5^Institute for Geology, Center for Earth System Research and Sustainability (CEN), Universität Hamburg, Hamburg 20146, Germany

^*^Corresponding author: [baoli.wang@tju.edu.cn](mailto:baoli.wang@tju.edu.cn) (B. Wang), Tel: +86 022 83614909, Fax: +86 022 27405053; Fushun Wang: [fswang@shu.edu.cn](mailto:fswang@shu.edu.cn) (F. Wang).

Address: 92 Weijin Road, Tianjin 300072 China

Emails of authors:

Baoli Wang: [baoli.wang@tju.edu.cn](mailto:baoli.wang@tju.edu.cn);

Cong-Qiang Liu: [liucongqiang@vip.skleg.cn](mailto:liucongqiang@vip.skleg.cn);

Stephen C. Maberly: [scm@ceh.ac.uk](mailto:scm@ceh.ac.uk);

Fushun Wang: [fswang@shu.edu.cn](mailto:fswang@shu.edu.cn);

Jens Hartmann: [geo@hattes.de](mailto:geo@hattes.de)

**1 Data collection**

The GEMS-GLORI world river discharge database is available online^1^ (<http://doi.pangaea.de/10.1594/PANGAEA.804574?format=html>) and 175 rivers with DSi concentration were selected. The names and locations of the 175 rivers are listed in Table 1. Data from the 2007 National Lakes Assessment (NLA) in the USA were available online (<http://water.epa.gov/type/lakes/NLA_data.cfm>), and 19 datasets were divided according to chlorophyll *a* concentration (Table 2) and the mean of each dataset was calculated and used. Data from the Changjiang River were derived from Chetelat *et al.*^2^

The GLORICH - Global River Chemistry Database^3^ is an update of the GEMS-GLORI database and couples river chemical information with catchment properties. The raw data from the GLORICH having DSi, HCO_3_^-^ and Na concentrations were used. In this study, the molar ratios of DSi/Na and HCO_3_^-^/Na were calculated.

**2 Survey of lakes in the English Lake District**

**Study sites**

The location of the 20 English lakes (Windermere is treated as two basins: North and South) and their geographical and physical features are reported in Table 3. Each lake was sampled at approximately its deepest point.

**Sampling**

Investigations were carried out in January, April, July, and October, 1991, 1995, 2000, 2005, and 2010. An integrated sample of surface water was taken using a weighted 5 m long plastic tube (7 m in Windermere). Replicate samples were dispensed into a previously rinsed 500 ml plastic bottle. After mixing thoroughly, water was decanted into: a) two disposable 500 ml plastic bottles, for nutrient analysis. b) a 500 ml plastic bottle containing 2.5 ml of Lugol’s iodine solution for subsequent enumeration and identification of algal populations^4^. The iodine was added to the algal cells to preserve them and increase their rate of sedimentation during subsequent processing in the laboratory. The remainder of the water sample was used for the determination of chlorophyll *a* concentration. A small glass bottle with a ground glass stopper was completely filled with lake water by submerging it just below the water surface and inserting the stopper so that no air was trapped within the bottle. This sample was used to determine the pH and alkalinity.

Temperature (T) and dissolved oxygen (DO) were determined *in situ* using several probes, which included a Wissenschaftlich-Technische Werstätten (WTW) Oxi 340i meter fitted with a combination thermistor and oxygen electrode (WTW TA197), a Hach HQd with LD0101 probe, and an YSI 6600 sonde.

**Nutrient and chemical analysis**

Nitrate, chloride, sulphate, sodium, calcium, magnesium and potassium concentrations were determined by ion chromatography using a Metrohm ion chromatograph. Dissolved reactive silica (DSi), alkalinity and pH were determined as described in Mackereth *et al.*^5^

**Algal pigments and populations**

The concentration of algal pigments was determined using the boiling methanol extraction procedure described by Vollenweider *et al*.^6^ A known volume of water was filtered through a Whatman GF/C filter, and pigments were extracted and analysed spectrophotometrically. A 300 ml sub-sample of the iodine-preserved water sample was concentrated to 5 ml by sedimentation. A known volume of the concentrated sample was transferred to a counting chamber and the algae were enumerated as described by Lund *et al.*^4^ Microplankton and nanoplankton were counted at 100× magnification and 400× magnification, respectively.

**Calculation of inorganic carbon speciation**

The DIC concentration in water comprises [CO_2_^*^] (free CO_2_ or dissolved CO_2_+ H_2_CO_3_) + [HCO_3_^-^] + [CO_3_^2-^]. Alkalinity, pH, and the appropriate temperature-dependent values of the dissociation constant were used to calculate equilibrium concentrations of CO_2_^*^, HCO_3_^-^, and CO_3_^2-^ according to Maberly^7^.

**Calculation of algal C and Si**

The biovolume of each species was geometrically calculated according to Hillebrand *et al.*^8^ Algal C and Si contents per cell were calculated on the basis of the relation between cell volume and cellular C and Si contents, respectively. The Si content of diatoms was calculated using the following regression equation (1) of Conley *et al.*^9^

log_10_ [Si content (pmol cell^-1^)] = 0.91 1og_10_ [biovolume (µm^3^)] - 3.16 (1)

The algal C content was calculated by the following regression equations (equation (2) for diatoms and equation (3) for other phytoplankton taxa) described by Menden-Deuer and Lessard^10^. The unit of C content (pg cell^-1^) was converted from atom weight to pmol cell^-1^.

log_10_ [C content (pg cell^-1^)] = 0.811 1og_10_ [biovolume (µm^3^)] – 0.541 (2)

log_10_ [C content (pg cell^-1^)] = 0.939 1og_10_ [biovolume (µm^3^)] – 0.665 (3)

The total C and Si amounts represented by each algal species were calculated according to their cell numbers and cellular C and Si contents. Finally, phytoplankton assemblage C and Si contents were obtained from the sums of the C and Si contents of the component species.

**3 Data analysis**

Data for the 175 rivers, American and English lakes are reported as average (Aver), standard deviation (SD), maximum (Max), and minimum (Min) (Tables 4 – 6). Pearson’s correlation coefficient was determined with the SPSS software (version 11.5; SPSS, Inc.), and results are in Tables 7 – 9.

Concentrations of dissolved chemicals were normalized to Na concentrations (Na-normalized) to eliminate the influence of water fluxes, water-rock interactions, and dilution/evaporation^11, 12^.

Characteristic molar ratios of end members in the river waters were estimated (Table 10). The end members for HCO_3_^-^/Na^+^ are from Gaillardet *et al.*^13^, the DSi/HCO_3_^-^ of carbonate-bedrock river water was calculated from the Wujiang River^14^, and the DSi/HCO_3_^-^ of continental weathering of silicate rocks is from Beaulieu *et al.*^15^ (equation 4).

4Na_0.5_Ca_0.5_Al_1.5_Si_2.5_O_8_ + 17H_2_O + 6CO_2_ → 3Al_2_Si_2_O_5_(HO)_4_ + 2Na^+^ + 2Ca^2+^ + 6HCO_3_^-^+ 4H_4_SiO_4_ (4)

**References**

1. Meybeck, M. & Ragu, A. GEMS-GLORI world river discharge database. *Laboratoire de Géologie Appliquée, Université Pierre et Marie Curie, Paris, France*, doi:10.1594/PANGAEA.804574 (2012).
2. Chetelat, B., *et al.* Geochemistry of the dissolved load of the Changjiang Basin rivers: anthropogenic impacts and chemical weathering. *Geochim. Cosmochim. Acta* **72**, 4254–4277 (2008).
3. Hartmann, J., Lauerwald, R., & Moosdorf, N. A brief overview of the global river chemistry database, GLORICH. *Procedia Earth Planet. Sci.* **10**, 23–27 (2014).
4. Lund, J. W. G., Kipling, C. & Le Cren, E. D. The inverted microscope method of estimating algal numbers and the statistical basis of estimations by counting. *Hydrobiologia* **11**, 143–170 (1958).
5. Mackereth, F. J. H., Heron, J., & Talling, J. F. Water analysis: some revised methods for limnologists (Vol. 36). *Ambleside: Freshwater Biological Association* (1978).
6. Vollenweider, R. A., Talling, J. F., & Westlake, D. F. A manual on methods for measuring primary production in aquatic environments. *IBP Handbook (IBP)* (1974).
7. Maberly, S. C. Diel, episodic and seasonal changes in pH and concentrations of inorganic carbon in a productive lake. *Freshw. Biol.* **35**, 579–598 (1996).
8. Hillebrand, H., Dürselen, C. D., Kirschtel, D., Pollingher, U. & Zohary, T. Biovolume calculation for pelagic and benthic microalgae. *J. Phycol.* **35**, 403–424 (1999).
9. Conley, D. J., Kilham, S. S. & Theriot, E. Differences in silica content between marine and freshwater diatoms. *Limnol. Oceanogr.* **34**, 205–212 (1989).
10. Menden-Deuer, S. & Lessard, E. J. Carbon to volume relationships for dinoflagellates, diatoms, and other protist plankton. *Limnol. Oceanogr.* **45**, 569–579 (2000).
11. Negrel, P., Allègre, C. J., Dupré, B. & Lewin, E. Erosion sources determined by inversion of major and trace element ratios and strontium isotopic ratios in river water: the Congo Basin case. *Earth Planet. Sci. Lett.* **120**, 59–76 (1993).
12. Gaillardet, J., Dupre, B., Allegre, C. J. & Négrel, P. Chemical and physical denudation in the Amazon River Basin. *Chem. Geol.* **142**, 141–173 (1997).
13. Gaillardet, J., Dupré, B., Louvat, P. & Allegre, C. J. Global silicate weathering and CO_2_ consumption rates deduced from the chemistry of large rivers. *Chem. Geol.* **159,** 3–30 (1999).
14. Han, G. & Liu, C. Q. Water geochemistry controlled by carbonate dissolution: a study of the river waters draining karst-dominated terrain, Guizhou Province, China. *Chem. Geol.* **204**, 1–21 (2004).
15. Beaulieu, E., Goddéris, Y., Labat, D., Roelandt, C., Oliva, P. & Guerrero, B. Impact of atmospheric CO_2_ levels on continental silicate weathering. *Geochem. Geophys. Geosyst.* **11**, Q07007 (2010).

**Tables**

**Table 1** Names and locations of the 175 rivers used in this study

| NO. | River | Country | Latitude | Longitude | NO. | River | Country | Latitude | Longitude |
| --- | --- | --- | --- | --- | --- | --- | --- | --- | --- |
| 1 | Adour | France | 43.32 | -1.32 | 89 | Mahanadi | India | 20.4333 | 85.9333 |
| 2 | Agano | Japon | 37.95 | 139.133 | 90 | Manavgat | Turkey | 36.7833 | 31.4333 |
| 3 | Altamaha | USA | 31.65 | -81.833 | 91 | Mekong | Vietnam | 9.7 | 106.3 |
| 4 | Amazon | Brazil | 0.1667 | -49 | 92 | Menjiang | China | 26.15 | 119.283 |
| 5 | Ameca | Mexico | 20.6833 | 105.3 | 93 | Mezen | Russia | 65.8333 | 44.3333 |
| 6 | Amguema | Russia | 68.1667 | -177.67 | 94 | Mississippi | USA | 28.95 | -89.4 |
| 7 | Amur | Russia | 53.1667 | 140.733 | 95 | Mobile | USA | 30.4833 | -88.017 |
| 8 | Anabar | Russia | 73.2333 | 113.583 | 96 | Mogami | Japon | 38.9167 | 139.8 |
| 9 | Anderson | Canada | 69.7167 | -128.97 | 97 | Moisie | Canada | 50.35 | -66.183 |
| 10 | Approuague | Guyana | 4.3333 | -52.117 | 98 | Murray | Australia | -35.367 | 139.367 |
| 11 | Bandama | Ivory Coast | 5.1667 | -5 | 99 | Musi | Indonesia | -2.3333 | 104.933 |
| 12 | Barito | Indonesia | -3.5333 | 114.483 | 100 | N. Dvina | Russia | 64.1 | 42.1667 |
| 13 | Bio Bio | Chile | -36.817 | -73.167 | 101 | Neches | USA | 30.35 | -94.1 |
| 14 | Brahmaputra | Bangladesh | 23.7 | 90.3667 | 102 | Negara | Indonesia | -3 | 114.75 |
| 15 | Brantas | Indonesia | -7.6333 | 112.733 | 103 | Negro | Argentina | -40.8 | -62.983 |
| 16 | Brazos | USA, Texas | 29.5833 | -95.75 | 104 | Nelson | Canada | 57.0667 | -92.5 |
| 17 | Bug | Ukraine | 47.55 | 30.7833 | 105 | Nemanus | Lithuania | 55.0333 | 21.8333 |
| 18 | Burdekin | Australia | -19.533 | 47.4167 | 106 | Neuse | USA | 35.1 | -76.5 |
| 19 | Bzyb | Georgia | 43.2 | 40.3 | 107 | Neva | Russia | 59.8 | 30.7167 |
| 20 | Cape Fear | USA | 33.8833 | -78 | 108 | Niger | Nigeria | 5.55 | 6.55 |
| 21 | Cauweri | India | 10.75 | 79.8333 | 109 | Nile | Egypt | 31.4333 | 31.8 |
| 22 | Cavally | Liberia | 4.3667 | -7.5333 | 110 | Nueces | USA | 28.0333 | -97.867 |
| 23 | Chang Jiang | China | 32.1 | 121.067 | 111 | Ob | Russia | 66.75 | 69.5 |
| 24 | Churchill | Canada | 58.7833 | -94.2 | 112 | Olenek | Russia | 72.9833 | 119.95 |
| 25 | Cimanuk | Indonesia | -6.3667 | 108.333 | 113 | Olfusa | Iceland | 64.05 | -21.167 |
| 26 | Citanduy | Indonesia | -7.6833 | 108.783 | 114 | Onega | Russia | 63.9667 | 37.9167 |
| 27 | Citarum | Indonesia | -5.9833 | 107.05 | 115 | Orange | South Africa | -28.683 | 16.4667 |
| 28 | Clutha | New Zealand | -46.35 | 169.8 | 116 | Orinoco | Venezuela | 8.6167 | -62.25 |
| 29 | Colorado | USA, Arizona | 32.7333 | -114.63 | 117 | Parana | Argentina | -34 | -58.283 |
| 30 | Colorado | USA, Texas | 29.3 | -96.1 | 118 | Pechora | Russia | 67.2 | 52.05 |
| 31 | Columbia | USA | 46.2 | -123.83 | 119 | Pee Dee | USA | 34.2 | -79.55 |
| 32 | Connecticut | USA | 41.9833 | -72.6 | 120 | Penobscot | USA | 44.8167 | -68.783 |
| 33 | Dalalven | Sweden | 60.6333 | 17.45 | 121 | Penzhina | Russia | 62.4667 | 165.3 |
| 34 | Danube | Rumania | 45.1833 | 28.8 | 122 | Po | Italia | 44.8833 | 11.65 |
| 35 | Daugava | Latvia | 56.8833 | 24.1333 | 123 | Potomac | USA | 38.9667 | -77.15 |
| 36 | Delaware | USA | 40.2167 | -74.783 | 124 | Progo | Indonesia | -7.8333 | 110.333 |
| 37 | Dnepr | Ukrainia | 46.5 | 32.3 | 125 | Purari | Papua New Guinea | -7.4167 | 145.083 |
| 38 | Dnestr | Ukrainia | 46.1667 | 30.3167 | 126 | Rhine | Nederland | 51.8667 | 6.0333 |
| 39 | Don | Russia | 47.25 | 39.75 | 127 | Rhone | France | 43.9167 | 4.6667 |
| 40 | Dongjiang | China | 23.1167 | 113.8 | 128 | Rio Grande | USA, Mexico | 25.8333 | -97.517 |
| 41 | Douro | Portugal | 41.15 | -8.6167 | 129 | Roini | Georgia | 42.1333 | 41.65 |
| 42 | Eastmain | Canada | 52.25 | -78.083 | 130 | Rogue | USA | 42.5833 | -124.07 |
| 43 | Ebro | Spain | 40.8167 | 0.5167 | 131 | Sabine | USA | 30.3 | -93.75 |
| 44 | Escambia | USA | 30.9833 | -87.3 | 132 | Sacramento | USA | 38.5833 | -121.5 |
| 45 | Fitzroy East | Australia | -23.367 | 150.533 | 133 | Saint John | Canada | 45.9667 | -67.233 |
| 46 | Flinders | Australia | -18 | 140.567 | 134 | Saint Lawrence | Canada | 46.8333 | -71.25 |
| 47 | Fraser | Canada | 49.3833 | -121.45 | 135 | Sakarya | Turkey | 40.75 | 30.3833 |
| 48 | Gambia | Gambia | 13.5167 | -14.833 | 136 | San Joaquin | USA | 37.6833 | -121.27 |
| 49 | Ganges | India | 24.0833 | 89.0333 | 137 | Sanaga | Cameroun | 3.7667 | 10.0667 |
| 50 | Garonne | France | 44.4167 | 0.2333 | 138 | Santee | USA | 33.45 | -80.15 |
| 51 | Gauja | Estonia | 27.15 | 24.2667 | 139 | Savannah | USA | 32.5333 | -81.267 |
| 52 | Godavari | India | 16.9167 | 81.7833 | 140 | Seal | Canada | 59.0667 | -94.8 |
| 53 | Gualdalquivir | Spain | 37.5167 | -5.9833 | 141 | Seine | France | 49.4333 | 0.4333 |
| 54 | Hanjiang | China | 23.3833 | 116.65 | 142 | Senegal | Senegal | 16.0167 | -16.5 |
| 55 | Hayes | Canada | 67.3 | -95.033 | 143 | Serayu | Indonesia | -7.6833 | 109.1 |
| 56 | Hong | Vietnam | 20.4167 | 106.2 | 144 | Shatt el Arab | Irak | 30.3333 | 48.25 |
| 57 | Huang He | China | 37.7333 | 118.6 | 145 | Shinano | Japan | 37.3 | 138.8 |
| 58 | Hudson | USA | 40.7 | -74.033 | 146 | Skeena | Canada | 54.6333 | -128.43 |
| 59 | Indigirka | Russia | 70.65 | 147.833 | 147 | Skeena | Canada | 54.6333 | -128.43 |
| 60 | Indus | Pakistan | 25.3833 | 68.4 | 148 | Solo | Indonesia | -6.7833 | 112.55 |
| 61 | Inguri | Georgia | 42.4 | 41.55 | 149 | Stikine | USA | 56.4667 | -132.38 |
| 62 | Irrawaddy | Myanmar | 15.75 | 94.8333 | 150 | Susitna | USA | 61.2667 | -150.5 |
| 63 | Ishikari | Japan | 43.25 | 141.383 | 151 | Susquehanna | USA | 40.25 | -76.867 |
| 64 | Jiulong | China | 24.4667 | 117.8 | 152 | Suwannee | USA | 29.95 | -82.933 |
| 65 | Kamchatka | Russia | 56.2333 | 162.467 | 153 | Tapti | India | 21.1667 | 72.9 |
| 66 | Kelantan | Malaysia | 5.7667 | 102.15 | 154 | Tejo | Portugal | 38.7333 | -9.1333 |
| 67 | Kennebec | USA | 44 | -69.833 | 155 | Tenryu | Japan | 34.65 | 137.783 |
| 68 | Khatanga | Russia | 72.9167 | 106 | 156 | Teshio | Japan | 44.8833 | 141.733 |
| 69 | Kikori | Papua New Guinea | -7.1667 | 144.083 | 157 | Thelon | Canada | 64.2667 | -96.083 |
| 70 | Kiso | Japan | 35.0333 | 136.75 | 158 | Thjorsa | Iceland | 63.7833 | -20.633 |
| 71 | Kitakami | Japan | 38.4167 | 141.317 | 159 | Tocantins | Brazil | -2.2 | -49.5 |
| 72 | Klamath | USA | 41.5167 | -123.97 | 160 | Tokachi | Japan | 42.7333 | 143.7 |
| 73 | Kobuk | USA | 66.75 | -161 | 161 | Tone | Japan | 35.7333 | 140.85 |
| 74 | Koksoak | Canada | 58.5333 | -68.167 | 162 | Tornionjoki | Finland | 65.8 | 24.1333 |
| 75 | Kola | Russia | 68.8833 | 33.0333 | 163 | Umpqua | USA | 43.7 | -124.05 |
| 76 | Kolyma | Russia | 68.5667 | 160.967 | 164 | Uruguay | Uruguay | -33.917 | -58.367 |
| 77 | Krishna | India | 16.5167 | 80.6167 | 165 | Venta | Latvia | 57.4 | 21.55 |
| 78 | Kuban | Russia | 45.2667 | 37.4 | 166 | Waikato | New Zealand | -37.283 | 175.067 |
| 79 | Kuskokwim | USA | 60.2833 | -162.45 | 167 | Waimakariri | New Zealand | -43.4 | 172.7 |
| 80 | Kymijoki | Finland | 60.5 | 26.8667 | 168 | Weser | Germany | 53.5333 | 8.5667 |
| 81 | La Grande | Canada | 53.7333 | -78.567 | 169 | Yana | Russia | 71.5167 | 136.533 |
| 82 | Lena | Russia | 70.7 | 127.65 | 170 | Yenisey | Russia | 71.8333 | 82.6667 |
| 83 | Lielupe | Latvia | 57.0167 | 23.9333 | 171 | Yodo | Japan | 34.8 | 135.633 |
| 84 | Limpopo | Mozambique | -25.25 | 33.5 | 172 | Yukon | USA | 62.65 | -164.8 |
| 85 | Loire | France | 47.2667 | -2.1833 | 173 | Zaire | Zaire | -6.0667 | 12.4 |
| 86 | Mackenzie | Canada | 68.2667 | -133.67 | 174 | Zambezi | Mozambique | -18.917 | 36.0667 |
| 87 | Magdalena | Columbia | 11.1 | -74.85 | 175 | Zhujiang | China | 22.6667 | 113.083 |
| 88 | Mahakam | Indonesia | -0.5833 | 117.283 |  |  |  |  |  |

**Table 2 Numbers of the data in different range of chlorophyll a (Chl *a*, μg l^-1^) in lakes of the United States of America**

| NO. | Range of Chl *a* | Numbers | NO. | Range of Chl *a* | Numbers | NO. | Range of Chl *a* | Numbers |
| --- | --- | --- | --- | --- | --- | --- | --- | --- |
| 1 | 1﹣2 | 141 | 8 | 8﹣9 | 28 | 15 | 60﹣70 | 29 |
| 2 | 2﹣3 | 123 | 9 | 9﹣10 | 26 | 16 | 70﹣80 | 16 |
| 3 | 3﹣4 | 98 | 10 | 10﹣20 | 177 | 17 | 80﹣90 | 14 |
| 4 | 4﹣5 | 70 | 11 | 20﹣30 | 108 | 18 | 90﹣100 | 11 |
| 5 | 5﹣6 | 63 | 12 | 30﹣40 | 65 | 19 | 100﹣200 | 69 |
| 6 | 6﹣7 | 47 | 13 | 40﹣50 | 42 |  |  |  |
| 7 | 7﹣8 | 38 | 14 | 50﹣60 | 28 |  |  |  |

**Table 3** Basic status of lakes in the English Lake District. (1): Catchment area (km^2^); (2): Mean catchment altitude (m); (3): lake length (km); (4): Max. width (km); (5): Area (km^2^); (6): Volume (m^3^×10^6^); (7): Mean depth (m); (8): Max. depth (m); (9): Approx. mean retention time (days); (10): Sampling location (NGR).

| Lake Name | (1) | (2) | (3) | (4) | (5) | (6) | (7) | (8) | (9) | (10) |
| --- | --- | --- | --- | --- | --- | --- | --- | --- | --- | --- |
| Bassenthwaite Lake | 360 | 333 | 6.2 | 1.1 | 5.3 | 27.9 | 5.3 | 19 | 30 | NY214296 |
| Blelham Tarn | 4.3 | 105 | 0.67 | 0.29 | 0.1 | 0.7 | 6.8 | 14.5 | 50 | NY366006 |
| Brothers Water | 13.2 | 437 | 0.6 | 0.4 | 0.2 | 1.5 | 7.2 | 15 | 21 | NY403127 |
| Buttermere | 18.7 | 377 | 2 | 0.54 | 0.9 | 15.2 | 16.6 | 28.6 | 140 | NY188154 |
| Coniston Water | 62.5 | 227 | 8.7 | 0.73 | 4.9 | 113.3 | 24.1 | 56.1 | 340 | SD298935 |
| Crummock Water | 62.7 | 327 | 4 | 0.85 | 2.5 | 66.4 | 26.7 | 43.9 | 200 | NY158192 |
| Derwent Water | 85.4 | 354 | 4.6 | 1.91 | 5.4 | 29 | 5.5 | 22 | 55 | NY267207 |
| Elterwater | 1 | 108 | 1 | 0.4 | 0.03 | 0.1 | 3.3 | 7 | 20 | NY329043 |
| Ennerdale Water | 43.5 | 374 | 3.8 | 1.1 | 3 | 53.2 | 17.8 | 42 | 200 | NY103153 |
| Esthwaite Water | 17 | 148 | 2.5 | 0.62 | 1 | 6.4 | 6.4 | 15.5 | 100 | SD358972 |
| Grasmere | 30.2 | 328 | 1.6 | 0.6 | 0.6 | 5 | 7.7 | 21.5 | 25 | NY340064 |
| Haweswater | 32.3 | 463 | 6.9 | 0.9 | 3.9 | 76.6 | 23.4 | 57 | 500 | NY478139 |
| Loughrigg Tarn | 0.95 | 175 | 0.4 | 0.3 | 0.07 | 0.5 | 6.9 | 10.3 | 117 | NY344044 |
| Loweswater | 8.2 | 243 | 1.8 | 0.55 | 0.6 | 5.4 | 8.4 | 16 | 150 | NY127215 |
| Rydal Water | 33.8 | 312 | 1.2 | 0.36 | 0.3 | 1.5 | 4.4 | 18 | 9 | NY358063 |
| Thirlmere | 53.8 | 398 | 6 | 0.78 | 3.3 | 52.5 | 16.1 | 46 | 280 | NY318154 |
| Ullswater | 147 | 393 | 11.8 | 1.02 | 8.9 | 223 | 25.3 | 63 | 350 | NY400190 |
| Wastwater | 42.5 | 385 | 4.8 | 0.82 | 2.9 | 115.6 | 40.2 | 76 | 350 | NY160058 |
| Windermere North Basin | 175 | 231 | 7 | 1.6 | 8.1 | 201.8 | 25.1 | 64 | 180 | NY383006 |
| Windermere South Basin | 250 | 231 | 9.8 | 1 | 6.7 | 112.7 | 16.8 | 42 | 100 | SD382914 |

**Table 4** Averages and range of major chemical variables in the 175 rivers. Unit of the variables are μmol l^-1^. Aver, Average; SD, standard deviation; Max, maximum; Min; minimum

|  | HCO_3_^-^ | SO_4_^2-^ | NO_3_^-^ | Cl^-^ | DSi | Ca^2+^ | Mg^2+^ | K^+^ | Na^+^ |
| --- | --- | --- | --- | --- | --- | --- | --- | --- | --- |
| Aver | 1295.6 | 277.5 | 8.2 | 686.6 | 165.3 | 588.9 | 298.4 | 55.9 | 774.2 |
| SD | 1091.6 | 498.0 | 12.6 | 2755.1 | 147.7 | 610.2 | 539.1 | 99.1 | 2163.3 |
| Max | 5262.3 | 3947.9 | 81.9 | 34781.4 | 1260.8 | 2709.6 | 6211.4 | 1074.2 | 24967.4 |
| Min | 52.5 | 5.2 | 0.2 | 8.5 | 1.7 | 20.0 | 14.8 | 5.1 | 8.7 |

**Table 5** Averages and range of major chemical variables in lakes of the United States of America. Units of chlorophyll *a* (Chl *a*) is μg l^-1^, and the others are μmol l^-1^. Aver, average; SD, standard deviation; Max, maximum; Min; minimum. N=1193

|  | HCO_3_^-^ | SO_4_^2-^ | NO_3_^-^ | Cl^-^ | DSi | Ca^2+^ | Mg^2+^ | K^+^ | Na^+^ | Chl a | DIC |
| --- | --- | --- | --- | --- | --- | --- | --- | --- | --- | --- | --- |
| Aver | 2355.4 | 2160.5 | 5.8 | 1602.5 | 143.5 | 714.5 | 1148.3 | 248.5 | 4115.5 | 23.6 | 2588.1 |
| SD | 3930.4 | 16234 | 23.0 | 14606 | 167.7 | 966.1 | 4808.5 | 1350.9 | 28191 | 34.7 | 4879.2 |
| Max | 63338 | 417293 | 386.2 | 446058 | 1521.8 | 12118 | 101461 | 36114 | 728150 | 198.7 | 77577 |
| Min | 1.5 | 1.25 | 0.1 | 1.5 | 0.4 | 5.9 | 6.5 | 1.3 | 10.3 | 1.0 | 18.0 |

**Table 6** Averages and range of major chemical variables in lakes of the English Lake District. Unit of chlorophyll *a* (Chl *a*) is μg l^-1^; and the others are μmol l^-1^. Aver, Average; SD, standard deviation; Max, maximum; Min; minimum. N=400

|  | HCO_3_^-^ | SO_4_^2-^ | NO_3_^-^ | Cl^-^ | DSi | Ca^2+^ | Mg^2+^ | K^+^ | Na^+^ | Chl a | DIC |
| --- | --- | --- | --- | --- | --- | --- | --- | --- | --- | --- | --- |
| Aver | 192.2 | 87.3 | 24.0 | 244.2 | 22.3 | 253.7 | 80.0 | 12.3 | 205.5 | 8.3 | 243.9 |
| SD | 122.7 | 26.4 | 13.7 | 78.2 | 11.6 | 124.1 | 24.1 | 6.3 | 49.3 | 10.8 | 137.6 |
| Max | 640.5 | 238.0 | 97.1 | 529.0 | 59.3 | 660.0 | 160.8 | 53.0 | 370.0 | 72.3 | 744.8 |
| Min | 8.0 | 6.0 | 1.2 | 101.0 | 0.7 | 73.0 | 35.0 | 4.3 | 120.0 | 0.3 | 34.7 |

**Table 7** Results of Pearson’s correlation coefficient analysis in the 175 rivers. N=175

|  | HCO_3_^-^ | SO_4_^2-^ | NO_3_^-^ | Cl^-^ | DSi | Ca^2+^ | Mg^2+^ | K^+^ |
| --- | --- | --- | --- | --- | --- | --- | --- | --- |
| SO_4_^2-^ | 0.608** |  |  |  |  |  |  |  |
| NO_3_^-^ | 0.432** | 0.390** |  |  |  |  |  |  |
| Cl^-^ | 0.291** | 0.572** | 0.602** |  |  |  |  |  |
| DSi | -0.060 | -0.092 | 0.024 | -0.028 |  |  |  |  |
| Ca^2+^ | 0.896** | 0.762** | 0.496** | 0.336** | -0.142 |  |  |  |
| Mg^2+^ | 0.559** | 0.690** | 0.580** | 0.921** | -0.062 | 0.530** |  |  |
| K^+^ | 0.247** | 0.420** | 0.643** | 0.815** | 0.091 | 0.246** | 0.744** |  |
| Na^+^ | 0.384** | 0.722** | 0.578** | 0.969** | -0.002 | 0.443** | 0.927** | 0.775** |
|  | HCO_3_^-^/Na^+^ | SO_4_^2-^/Na^+^ | NO_3_^-^/Na^+^ | Cl^-^/Na^+^ | DSi/Na^+^ | Ca^2+^/Na^+^ | Mg^2+^/Na^+^ |  |
| SO_4_^2-^/Na^+^ | 0.619** |  |  |  |  |  |  |  |
| NO_3_^-^/Na^+^ | -0.101 | -0.038 |  |  |  |  |  |  |
| Cl^-^/Na^+^ | 0.446** | 0.516** | 0.088 |  |  |  |  |  |
| DSi/Na^+^ | 0.473** | 0.436** | -0.264** | 0.297** |  |  |  |  |
| Ca^2+^/Na^+^ | 0.966** | 0.698** | -0.074 | 0.446** | 0.419** |  |  |  |
| Mg^2+^/Na^+^ | 0.914** | 0.757** | -0.077 | 0.541** | 0.471** | 0.914** |  |  |
| K^+^/Na^+^ | 0.373** | 0.445** | -0.116 | 0.780** | 0.482** | 0.359** | 0.387** |  |

** Correlation is significant at p <0.01 (2-tailed); *correlation is significant at p<0.05 (2-tailed).

**Table 8** Results of Pearson’s correlation coefficient analysis in lakes of the United States of America. N=1193

|  | HCO_3_^-^ | SO_4_^2-^ | NO_3_^-^ | Cl^-^ | DSi | Ca^2+^ | Mg^2+^ | K^+^ | Na^+^ | Chl a |
| --- | --- | --- | --- | --- | --- | --- | --- | --- | --- | --- |
| SO_4_^2-^ | 0.491** |  |  |  |  |  |  |  |  |  |
| NO_3_^-^ | 0.008 | -0.013 |  |  |  |  |  |  |  |  |
| Cl^-^ | 0.224** | 0.184** | -0.009 |  |  |  |  |  |  |  |
| DSi | 0.279** | -0.022 | 0.031 | -0.016 |  |  |  |  |  |  |
| Ca^2+^ | 0.153** | 0.339** | 0.025 | 0.215** | 0.123** |  |  |  |  |  |
| Mg^2+^ | 0.440** | 0.907** | -0.012 | 0.301** | 0.028 | 0.440** |  |  |  |  |
| K^+^ | 0.764** | 0.692** | -0.010 | 0.301** | 0.138** | 0.099** | 0.586** |  |  |  |
| Na^+^ | 0.611** | 0.908** | -0.013 | 0.517** | -0.008 | 0.260** | 0.809** | 0.796** |  |  |
| Chl a | 0.064* | 0.056 | -0.009 | 0.002 | 0.233** | 0.130** | 0.060** | 0.092* | 0.046 |  |
| DIC | 0.990** | 0.485** | -0.001 | 0.242** | 0.257** | 0.121** | 0.417** | 0.789** | 0.621** | 0.067* |

** Correlation is significant at p <0.01 (2-tailed); *correlation is significant at p<0.05 (2-tailed).

**Table 9** Results of Pearson’s correlation coefficient analysis in lakes of the English Lake District. N=400

|  | HCO_3_^-^ | SO_4_^2-^ | NO_3_^-^ | Cl^-^ | DSi | Ca^2+^ | Mg^2+^ | K^+^ | Na^+^ | Chl a |
| --- | --- | --- | --- | --- | --- | --- | --- | --- | --- | --- |
| SO_4_^2-^ | 0.451** |  |  |  |  |  |  |  |  |  |
| NO_3_^-^ | 0.075 | 0.390** |  |  |  |  |  |  |  |  |
| Cl^-^ | 0.225** | 0.580** | 0.358** |  |  |  |  |  |  |  |
| DSi | -0.266** | -0.116* | 0.259** | -0.109* |  |  |  |  |  |  |
| Ca^2+^ | 0.906** | 0.646** | 0.242** | 0.487** | -0.270** |  |  |  |  |  |
| Mg^2+^ | 0.687** | 0.740** | 0.263** | 0.618** | -0.173** | 0.787** |  |  |  |  |
| K^+^ | 0.670** | 0.757** | 0.386** | 0.624** | -0.149* | 0.795** | 0.796** |  |  |  |
| Na^+^ | 0.371** | 0.665** | 0.344** | 0.927** | -0.124* | 0.613** | 0.696** | 0.716** |  |  |
| Chl a | 0.572** | 0.172** | -0.140** | 0.144** | -0.404** | 0.528** | 0.396** | 0.390** | 0.227** |  |
| DIC | 0.967** | 0.461** | 0.148** | 0.274** | -0.136** | 0.893** | 0.677** | 0.673** | 0.403** | 0.525** |

** Correlation is significant at p <0.01 (2-tailed); *correlation is significant at p<0.05 (2-tailed).

**Table 10** Characteristic molar ratios of the end members in the river waters.

|  | HCO_3_^-^/Na^+^ | DSi/HCO_3_^-^ | ^87^Sr/^86^Sr |
| --- | --- | --- | --- |
| Carbonate-bedrock | ～120^a^ | ～0.036^b^ | ～0.708 |
| Silicate-bedrock | ～2^a^ | ～0.67^c^ | ～0.725 |

^a^ From Gaillardet *et al*.^13^; ^b^ From Han and Liu^14^; ^c^ From Beaulieu *et al*.^15^
